# A Bibliometric Analysis of Telephone Triage Research to 2021 Using VOSviewer

**DOI:** 10.1155/2024/5583853

**Published:** 2024-05-28

**Authors:** Jill Poots, Jim Morgan, Matteo Curcuruto

**Affiliations:** School of Humanities and Social Sciences Leeds Beckett University, Leeds, England, UK

## Abstract

Telephone triage services are becoming increasingly commonplace in modern healthcare. Despite this widespread adoption, health researchers and practitioners seeking to understand evidence-based best practice face several challenges. Firstly, the few systematic reviews available yield small sample sizes, suggesting a small amount of research. Secondly, the rapid pace of development of telephone triage technologies means that there may be temporal validity issues with the available research, given some of this research is relatively old. Thirdly, researchers use different terminologies to describe telephone triage, meaning evidence may be more difficult to find than if consistent terminology was used. This bibliometric analysis therefore is aimed at providing a macroscopic overview of telephone triage, to understand the scale and scope of the available evidence (i.e., where, when, and by whom research is conducted), for interested researchers and practitioners. Additionally, it is aimed at quantifying the prevalence of terms used to describe telephone triage, to recommend consistent terminology for future use, and to improve accessibility of research. To address these aims, literature searches using three different key terms: “telephone triage,” “remote triage,” and “teletriage” were conducted in Scopus and PubMed. Corresponding bibliometric data was visualised and analysed using VOSviewer. This bibliometric review identified 784 papers since the term “telephone triage” first appears in 1980, confirming a paucity of literature in the field. An overview of telephone triage research up to 2021 is provided, which should serve as a useful foundation for future research and application of evidence-based practice. Gaps in telephone triage research are identified, and the use of consistent terminology is encouraged, with the aim of supporting telephone triage researchers in determining research priorities and improving the impact of future studies.

## 1. Introduction

Telemedicine, “the use of electronic information and communications technologies to provide and support health care when distance separates the participants,” [1] use is growing. Telephone triage is a telemedicine activity which assesses a caller's symptoms via telephone. These symptoms can range from minor (e.g., cold symptoms) to highly urgent (e.g., breathing difficulties and chest pain). The caller is then directed to appropriate healthcare based on the urgency of their needs [2]. Telephone triage was originally used for settings where visits from medical professionals were obstructed, e.g., after natural disasters [3]. However, since the 1990s, telephone triage has become increasingly widespread due to greater availability of communication technology, the introduction of out-of-hours (OOH) services, and the redistribution of triage work to nurses (1). In the early 2000s, several countries scaled up telephone triage activity, introducing national telephone triage hotlines based in call centres, for example, NHS 111 in the UK (a successor of NHS Direct), Healthcare Direct in Sweden, Healthdirect Australia, and various “nurse lines” in the USA [4, 5]. These serve as a “front door” to wider health services, offering advice to patients who are uncertain as to the most appropriate support available to them [4]. Recently, telephone triage usage accelerated due to social distancing measures imposed to curb the spread of the COVID-19 virus, which made telephone triage essential to protect both healthcare professionals and the clinically vulnerable [6]. Call volumes for some services have not yet fallen to prepandemic levels [7], which suggests that telephone triage usage is not only widely accepted for social distancing purposes but is now standard healthcare practice, perhaps due to its convenience and high patient satisfaction levels [4, 7, 8].

Though popular in use, researchers and practitioners interested in telephone triage research are met with several challenges. Despite the steady long-term growth, recent widespread adoption, and anticipated continuance of telephone triage use, relatively little evaluative research emerges from database searches, and several systematic reviews have yielded small sample sizes of studies [9, 10], suggesting a paucity of research. This could also be due to inconsistent terminology used by researchers. Preliminary searches by the lead author revealed variations in the terminology for telephone triage, for example, “teletriage” (and its spelling variants) or “remote triage.” Conflicting terminology could make relevant research more difficult to identify for the purposes of reviews and determining evidence-based practice. Therefore, researchers should consider which key terms will increase the discoverability and impact of future research and move towards using consistent terminology to make comprehensive literature searches easier. Reviews exploring and comparing the use of terminologies would help to understand the most common terminologies and help increase discoverability of research through advocating for consistent language use.

Systematic reviews of telephone triage have stated that existing available research varies in both research quality and overall findings, making it difficult to interpret findings to inform evidence-based practice. Telephone triage is used in a variety of settings, from primary (e.g., GP surgeries) to tertiary (e.g., before scheduled care) healthcare settings. There are differences between the scale of operations, ranging from small general practitioner's offices to large-scale call centre operations. Moreover, some organisations employ nonclinical staff to triage (e.g., England's NHS 111 and Belgium's 1733), whilst others use exclusively clinical staff [11]. Services also differ in their accessibility, offering telephone triage universally or exclusively to specific populations, such as privately insured populations or employees [12, 13]. These discrepancies in service design could account for the variation in research findings, and researchers should consider what evidence is applicable to their individual settings. Moreover, a systematic review published in 2011 suggested that telephone triage research may be outdated [9]. With more recent advances in the development and availability of communications and clinical support technology (e.g., electronic care records and computer decision support systems), researchers must consider temporal validity issues before interpreting and applying the findings of older research to their settings.

Due to the challenges outlined above, it is proposed that a summary of the available research and corresponding gaps would be useful for those with an interest in telephone triage. Such a summary would ensure that those interested would be able to ascertain what works, where and for whom, in order to establish and apply evidence-based practices in their specific settings, to improve care quality. Moreover, it could enable researchers to identify opportunities for more detailed, systematic reviews of specific telephone triage settings and/or outcomes, as well as the requirements for further empirical research (i.e., research gaps), to ensure that patients are appropriately assessed by telephone. An additional aim of this review is to identify the most common and, therefore, most discoverable key terms in the research to help future telephone triage researchers to increase the discoverability and, consequently, the impact of their research.

To present an overview of the research literature, bibliometric analysis will be employed. Bibliometric analysis uses citation information to provide a summary of research in a particular field. Citation information may include author names, countries, and institutions; journal information; and keywords used in research articles. Collaboration information and overarching themes can also be identified through network mapping using specialist software. Bibliometric analysis can both quantify the prevalence of research terminologies and provide a macroscopic overview of an entire field, which can complement more granular systematic reviews and identify gaps for further empirical study [14]. These attributes make it a suitable method for the current research.

In summary, the current bibliometric analysis will provide a high-level overview of telephone triage research by addressing the following research questions:

1. How many research articles pertaining to telephone triage have been published?
2. What is the most widely used research terminology for telephone triage?
3. Where has telephone triage research taken place and by whom?
4. What are the main journals reporting on telephone triage research?
5. What are the most dominant topics in the literature corpus, according to keywords?

Citation data will be analysed using VOSviewer 1.6.17 [15]. VOSviewer software visualises citation information, using “cluster mapping” to identify collaboration between author groups, countries, and keywords. The resulting overview of the scale and scope of research can be used by researchers, practitioners, and policymakers involved in telephone triage operations. This analysis also complements a more focused systematic review on telephone triage safety [16]. Unlike systematic reviews, no standardised reporting framework exists for bibliometric analysis; therefore, the methods are described transparently, to increase the accessibility of the method for interested researchers.

## 2. Materials and Methods

### 2.1. Database Searching

A preliminary narrative review [17] revealed variations in telephone triage terminology used in research. This informed the search strategies for the current review. Initial searches were performed in PubMed and Scopus databases since these contain large volumes of both medical and multidisciplinary research literature. Scopus records detailed bibliometric information (e.g., country of affiliation), making it particularly appropriate to address most of the aims of the current review. The exception to this was keyword information. PubMed uses Medical Subject Headings (MeSH) to index keywords, which are standardised and therefore more consistent than author or automatically generated keywords. For this reason, PubMed was used for additional keyword analysis to better understand the topics within telephone triage research stored in this database.

To include as many publications as possible and understand the prevalence of different terminology, search terms describing telephone triage by its alternative names and spellings (i.e., teletriage and remote triage) were used to find articles of interest. This method has been used successfully to understand the trends in conflicting terminologies relating to the broader topic of telehealth [18]. Terms such as “virtual triage” or “e-triage” were excluded as these are most commonly associated with websites or applications [19, 20]. Given the interest of the current analysis in human patients, veterinary science documents were excluded from the searches. Considering the aims of the study, the following search strands were input to Scopus in December 2021:

1. All variations of key terminology together to capture as many publications as possible, i.e., [telephone triage] OR [telephone-triage] OR [teletriage] OR [tele-triage] OR [tele triage] OR [remote triage] AND (EXCLUDE(SUBJAREA, “VETE”))
2. Separate searches for the three main terminologies (telephone triage, teletriage, and remote triage) and their variations, e.g., [tele triage] OR [tele-triage] OR [teletriage] AND (EXCLUDE(SUBJAREA, “VETE”))

The use of brackets ensures that the search results yielded the precise terminology. Since this is an exploratory bibliometric analysis aimed at scoping out research trends and productivity, no further parameters linked to publication year, discipline, document type, or journal were utilized, which is common in exploratory bibliometric analyses, to provide an overview of research in a particular field [18, 21].

The bibliometric information considered useful for the current analyses included the following: author name(s), title, publication year, document type, journal, abstract, keywords, and author affiliations. The first search combining all key terminologies yielded 785 documents. The corresponding citation information for all articles was downloaded as a comma separated value (CSV) file in December 2021, for analysis in Microsoft Excel. One publication which had a date in the future year 2022 was excluded from the CSV to avoid skewing the research trends due to incomplete data for that year. This means that the total number of articles taken forward for further analyses was 784 (see Table 1). This sample is slightly larger than for PubMed (*n* = 679), which could reflect Scopus' inclusion of subjects PubMed does not cover, such as agriculture and astronomy.

### 2.2. Screening and Organising Citation Information

The downloaded CSV files containing the four separate searches (one for all variations in terminology and three pertaining to each specific terminology) were analysed using Microsoft Excel. To generate an understanding of the trends for the whole field and avoid duplications, the three separate searches were combined into one spreadsheet, and exact duplicates were removed by manual screening by the lead researcher. Duplicates included papers which used multiple terminologies (e.g., teletriage and telephone triage might be mentioned in one paper). Abstracts and titles were screened, and duplicate papers were assigned to a group according to the terminology which was most prevalent in the abstract. CSV files were also used for the visualisation of similarity (VOS) mapping using VOSviewer [22]. In line with recommendations from van Eck and Waltman [15], a thesaurus file was created to correct any anomalies due to misspelled locations or author names which may affect the accuracy of the mapped outputs (e.g., “AustraliaWA” became “WA, Australia”). At this point, papers were screened to only include peer-reviewed research outputs (i.e., journal articles, reviews, and conference papers). Letters, commentaries, addendums, and news articles were excluded, leaving 724 articles remaining in the Scopus sample.

### 2.3. Bibliometric Analyses

Microsoft Excel was used to analyse and chart as follows:

1. Document types (e.g., conference paper and journal article)
2. Number of publications per year
3. Most cited authors and journals

CSV files were input to VOSviewer for further analysis. VOSviewer can group related items (e.g., keywords and author names) together in “clusters.” Three types of maps can be created: network, density, and overlay. These mapping functions were used to analyse as follows:

1. Countries and their relationships
2. Most cited authors and collaborating authors
3. Themes emerging from the literature using keywords
4. Research trends over time (using cluster analysis of keywords)

### 2.4. Interpretation of VOSviewer Maps

**Figure 1:**
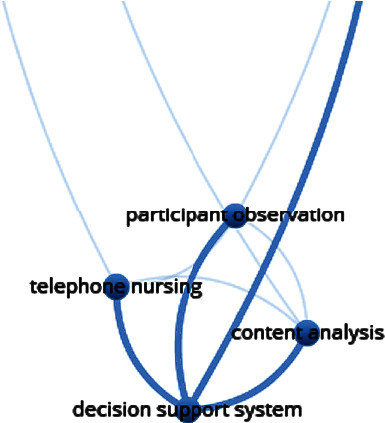
Guide to interpreting the bibliometric map in VOSviewer.

Clusters of linked items appear in the same colour and are numbered for interpretation. Connections between items, such as the co-occurrence of keywords or coauthorship between researchers, are represented by a “link” (indicated by a line connecting two items). A stronger link, for example, indicates that two items have more publications in common and will have a higher numerical value. The bigger the item, the more it is cited in the research. The closer the distance between two items, the stronger the relationship between them. A detailed example is provided in Figure 1.

## 3. Results

Findings relating to trends in the overall research, terminology use, journals utilized, paper citation frequency, and countries producing research (including collaborations) will be presented. In addition, keyword themes will also be described in this section. Methodological considerations will be discussed briefly where relevant. As previously outlined in Materials and Methods and unless stated otherwise, findings reflect all search terms combined and retrieved from the Scopus database. MeSH keywords were identified by PubMed.

### 3.1. Trends in Telephone Triage Research over Time

**Figure 2:**
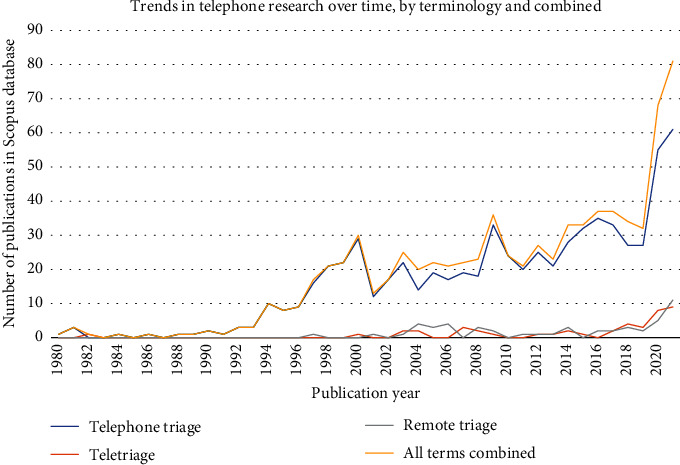
Trends in research over time, per terminology, and all terminology combined.

Figure 2 shows the trends in research output over time. The earliest published literature identified in Scopus dated from 1980, which is consistent with the PubMed database. There was minimal research published pertaining to telephone triage throughout the 1980s, with a median of 1 paper published per year. Research output increased in the 1990s and 2000s. The median number of papers published in these two decades was 8 and 17.5, respectively. More recently, there has been a rapid increase in telephone triage publications. Between 2019 and 2020, the number of published research articles doubled from 30 to 60, respectively. By the time of writing (December 2021), 71 papers pertaining to telephone triage had been published in 2021, suggesting that research output is continuing to rise.

### 3.2. Trends in Terminology Use

The searches pertaining to each terminology were analysed separately to understand trends in usage. Figure 2 shows similar trends in the use of all three terms, whereby after a period of steady growth, there has been a rapid increase in recent years, most notably in research using the term “telephone triage.” In 2021, sixty-one papers were published including this term. This is greater than any other year and almost double the highest documented figure (*n* = 35) in 2016. As shown in Table 2, “telephone triage” was the most prevalent term, followed by “remote triage” and “teletriage.” “Telephone triage” and “teletriage” first appeared in research literature in the early 1980s (though “teletriage” did not appear again until 2000). Despite this, there is a large difference between the volume of research for both terms, with only 5.5% of the total volume of published research pertaining to “teletriage.” “Remote triage” is a relatively new term (first used in 1997) yet is slightly more prevalent in the literature than “teletriage,” suggesting that “teletriage” has been a less popular term in recent years.

### 3.3. Journals Publishing Telephone Triage Research and Article Citation Frequencies

The search yielded research from 162 peer-reviewed sources.  Table 3 shows journals with more than ten publications. The Journal of Telemedicine and Telecare was the journal with the most publications; however, this number was still small (*n* = 18) compared to the entire sample (*n* = 784). This indicates that there is a wide range of journals publishing telephone triage research. Whilst many of these journals were related to general practice or healthcare, some relate to specialist fields, such as pediatrics and oncology, suggesting that telephone triage is used in a variety of healthcare settings.

Table 4 shows the ten most cited journal articles. Bunn et al.'s [23] review paper on the effect of telephone triage on healthcare use and satisfaction is the most cited publication, with 180 citations. Some papers merely mention telephone triage, though it is not the subject of the research, for example, Diehr et al.'s [24] paper, which advocates for the use of telephone triage when treating acute cough features (158 citations). This highlights a limitation of bibliometric analysis: that papers featuring the keywords will be synthesised despite not being the sole focus of the research. Moreover, this suggests that the available research literature focusing on telephone triage is potentially even rarer than the review suggests, making the case for further research.

### 3.4. Countries Participating in Telephone Triage Research

**Figure 3:**
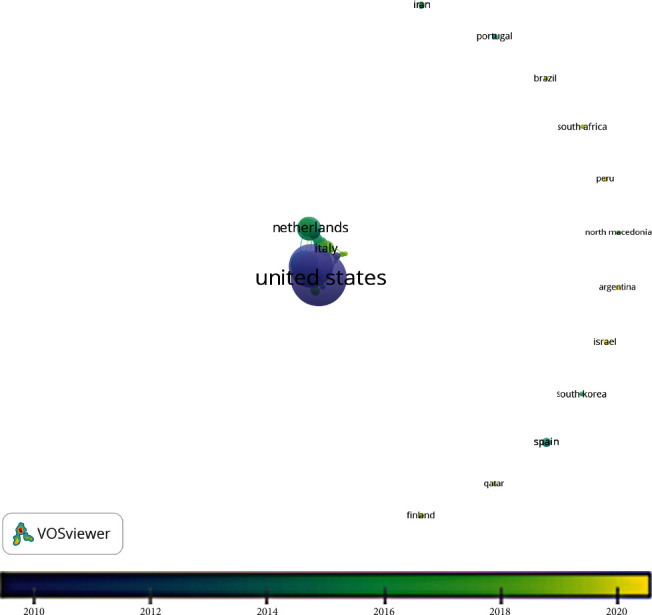
Overlay map showing all countries conducting telephone triage research, in recent years.

**Figure 4:**
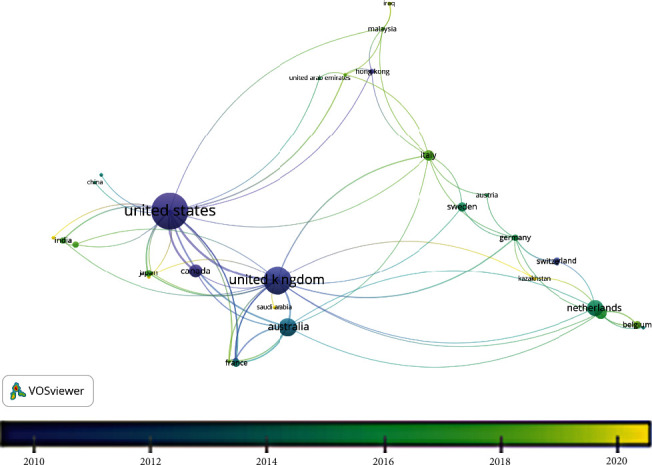
Zoomed view of cluster map showing strongest cluster of countries coauthoring telephone triage research.

VOSviewer uses affiliation data to provide an overview of countries producing research in a particular field. The authors from forty-four countries were found to have published telephone triage research, shown in Figure 3. The most tightly clustered countries (indicative of strong collaboration links) are shown in Figure 4.

Most research was published by European (45%) and North American (38%) researchers as outlined in Table 5.

The ten countries publishing the most telephone triage research are listed in Table 6. The United States produced the most publications (*n* = 263), followed by the United Kingdom (*n* = 160) and Australia (*n* = 63). The United States, United Kingdom, and Canada were the first to publish telephone triage research (see Figure 3). Most research took place in developed, westernised countries. However, more recently, research has been undertaken in South America and the Middle East (Figure 4). South America, Africa, and Asia are under-represented in telephone triage research, which could be due to the lack of availability of telephone triage services in these countries or could be a limitation of the Scopus search. All searches were conducted in the English language, meaning search output consisted primarily of articles published in English or with English translations in titles and abstracts.

**Figure 5:**
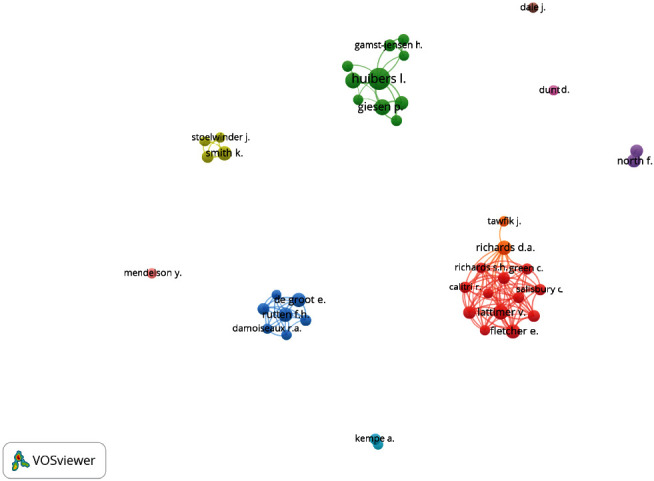
A cluster map showing authors coauthoring papers in telephone triage research.

Figure 5 contains the VOSviewer output of a cluster analysis showing the co-occurrences of authors with more than five occurrences in the dataset. Three larger clusters of coauthorship can be identified for authors. On closer inspection of the articles, these clusters seem to represent country-specific research, with UK researchers presented in red, Danish researchers in green, and Dutch research groups in blue. There is limited collaboration between these groups, suggesting that research is often system-specific, for example, within specific triage settings such as national telephone triage services or out-of-hours cooperatives. Future research could benefit from cross-country explorations and validation to share learning and best practice. There are some limitations to using VOSviewer for this purpose; for example, the country information is linked to the affiliations of researchers who may research outside their country of work. Author data (Figure 5) also provides an overview of collaborations between countries.

### 3.5. Understanding Research Themes according to Using Keyword Analysis

**Figure 6:**
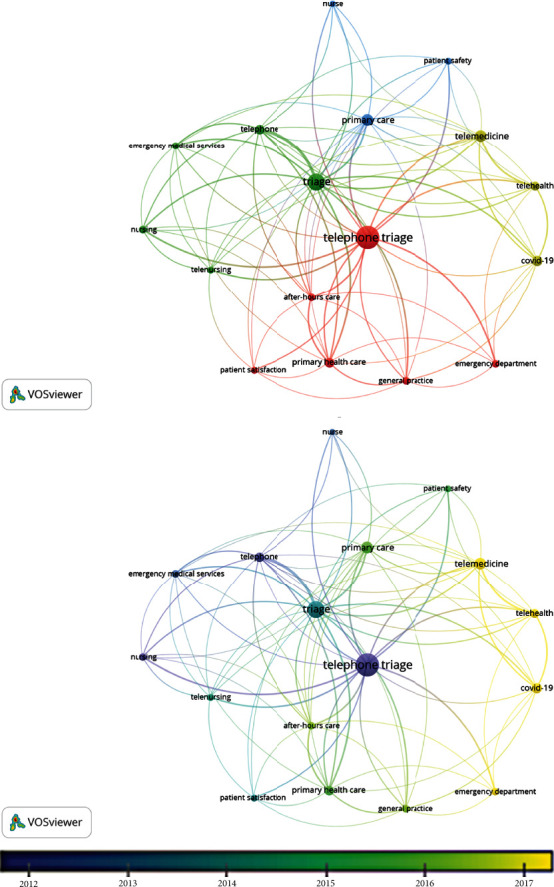
Overlay and cluster maps for author-indexed keywords with more than ten occurrences.

VOSviewer can use natural language processing algorithms, such as noun phrase identification, to extract relevant key terms from the available bibliometric data (i.e., the “all key terms” function), as well as author-indexed keywords. Many key terms were generated (*n* = 3718 for all key terms and *n* = 1118 for only author-indexed key terms). To increase accuracy, author-indexed key terms were used to understand research trends. Additionally, the map was restricted to contain only keywords occurring more than ten times. This yielded a smaller but more manageable sample of keywords (*n* = 17, Figure 6), which helped to ascertain the most popular author keywords in recent years. Aside from the main search terms “telephone triage” (*n* = 139) and “triage” (*n* = 78), the most frequently occurring author keywords were “primary health care” and “telemedicine,” each with 35 occurrences, and “Covid-19,” which had 29 occurrences. “Covid-19,” “telemedicine,” and “telehealth” were the most recent prevalent key terms. Generally, commonly occurring key terms describe the setting, and only two keywords describe the outcome being investigated (i.e., “patient satisfaction” and “patient safety”).

Coloured clusters of keywords in cluster maps show co-occurring keywords, which can offer insights into the themes in the research. The cluster map in Figure 6 displays four clusters of keywords, which could be described as follows: telenursing studies (green), patient safety in primary care studies (blue), studies relating to telemedicine in COVID-19 (yellow), and patient satisfaction in primary and emergency care (red). There are some caveats with this method of identifying research themes, such as the need for subjective interpretation by the analyst and discrepancies in keywords used by authors (e.g., “primary care” and “primary health care” are separate items appearing in two different clusters, despite likely referring to the same telephone triage setting). However, the large variety of key terms identified in only a small sample of publications captured may be interpreted as reflecting great diversity in telephone triage research contexts, aims, and variables. To improve consistency, controlled lists of keywords, such as Medical Subject Headings (MeSH), SNOMED CT, or ICD-10, could be used by authors to generate keywords. This may increase the discoverability and improve the indexing of their work. The potential for this is explored below.

**Figure 7:**
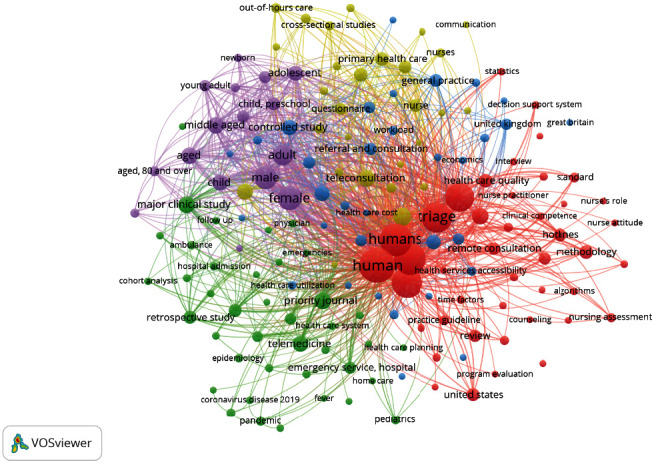
A cluster map showing the most prevalent key terms generated by VOSviewer using the “all keywords” function.

To interrogate the body of research further and give a broader overview of research themes, a map of co-occurring key terms was created using the “all keywords” function (Figure 7). To reflect the larger sample and understand the most prevalent key terms, the analysis was limited to keywords with more than twenty occurrences in the literature. Despite restricting the keywords to those with high occurrences, there was a lot of “noise” in the map due to database indexing, a consideration for future researchers. For example, the most commonly occurring keywords were “human” and “humans,” with 559 and 470 occurrences, respectively, likely reflecting indexed keywords in the database. The next most commonly occurring keyword was “emergency health service” (*n* = 469), followed by keywords pertaining to demographics. Once again, five thematic clusters were interpreted according to the key terms within them, and these might reflect the following particulars of the research:

1. Key terms relating to specific patient demographics (purple)
2. Key terms relating to emergency care and pandemics (green)
3. Key terms relating to the United Kingdom's primary care system (blue)
4. Key terms relating to methods and outcomes (red)
5. Key terms relating to research methods, which may reflect the evaluation of services such as pilot studies (yellow)

There was much overlap between clusters, indicating that many of the key terms are closely related, most likely due to the common indexed key terms “human(s)” and “triage.”

The text file from the bibliometric analysis, which included keywords appearing more than twenty times, was used to further identify keywords pertaining to health professionals performing triage, settings, and research outcomes. These keywords are shown in Table 7. Similar terms were merged and are indicated by “/” and “()” in the table.

The most commonly occurring healthcare setting was emergency care, with 625 keyword occurrences. Keywords pertaining to healthcare personnel referred to staff with clinical training. Quality was the most common keyword pertaining to outcomes of investigation, followed by safety.

### 3.6. Using Medical Subject Headings (MeSH) Keywords to Validate Themes

**Figure 8:**
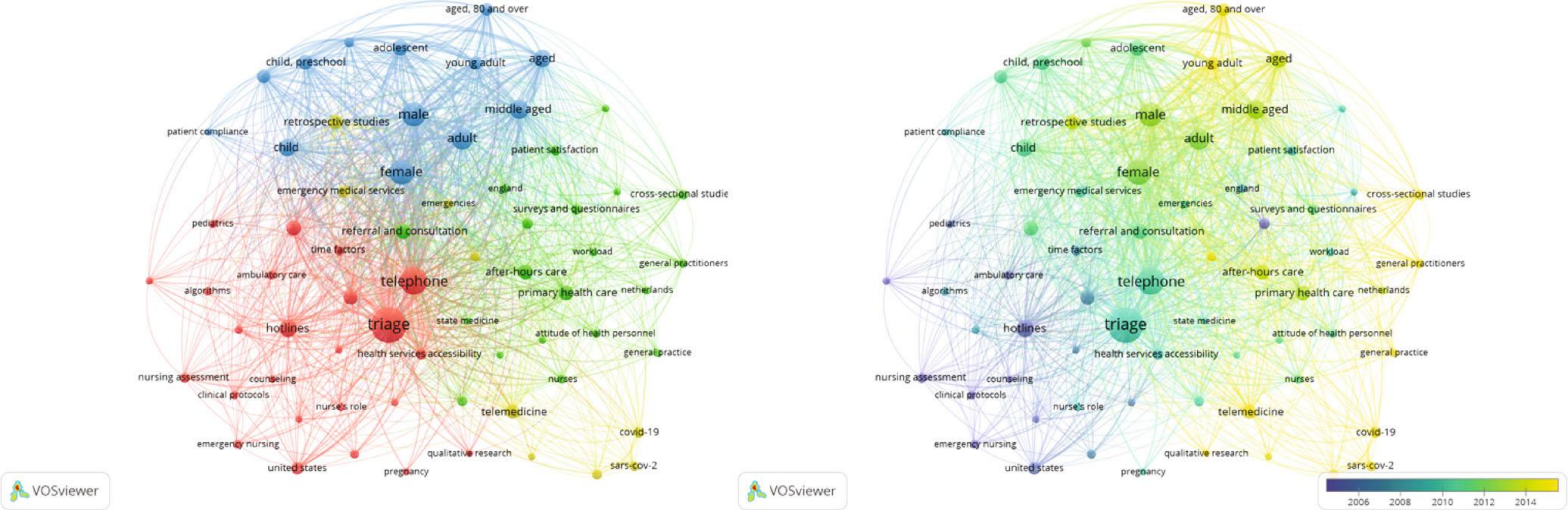
Overlay and cluster maps for MeSH key terms retrieved from PubMed searches.

Due to the subjectivity in interpreting author- and software-generated keywords from thematic clusters in VOSviewer, a PubMed search was performed to identify MeSH keywords and compare the clusters of keywords with more than twenty occurrences in this database (Figure 8). The aim was to validate keyword findings in Scopus. MeSH keywords are controlled and taxonomized into a hierarchical structure; therefore, it is anticipated that this would make the keyword analysis more objective. Similar clusters of keywords were identified using PubMed-generated MeSH key terms, to those generated by Scopus. Generated clusters included a cluster of keywords relating to patient demography (blue), a cluster of keywords relating to measures in primary care (green), a cluster relating to telephone nursing (red), and a cluster of key terms relating to telemedicine in pandemics such as COVID-19. The overlay view was used to understand the changes in the use of these key terms over time. The key term information provides an overview of the research settings which telephone triage research has focused on over time. Research pertaining to nursing hotlines, ambulatory care, pediatrics, and family practice emerged first in the literature before general practitioners and after-hours. The term “telemedicine” appeared more recently, along with “COVID-19” and “SARS-COV-2,” likely accounting for the rise in published research more generally.

## 4. Discussion

The overarching aim of the current bibliometric analysis was to provide an overview of the telephone triage literature, including abundance, trends, terminologies, and research themes. This information is intended to serve as a useful starting point for researchers and practitioners seeking to decipher evidence-based practice, direct future research endeavours, and increase the discoverability of their research through the use of appropriate and consistent terminology.

The first aim was to understand the abundance of telephone triage research. Over a forty-year period, fewer than 800 peer-reviewed articles relating to telephone triage were identified in Scopus (*n* = 784) and PubMed (*n* = 679). This relatively small number of articles suggests that telephone triage is a somewhat neglected area of healthcare research. Considering the widespread adoption of telephone triage, with millions of callers triaged per month in some systems, e.g., NHS 111 [7], more research is likely needed to understand and implement best practice.

Analysis of keyword and trend data enabled an interpretation of historical research trends. Other than a slight decrease in research output in 2001, there was an increasingly upward trend in the use of “telephone triage” from 2000 to 2019. The decrease in 2001 may reflect a decrease in research outputs related to the telemedicine “industry” more generally, the reasons for which are unknown [18].

Generally, the corpus of research literature has grown steadily since the 1990s. Previous authors have suggested the slight rise in the 1990s likely reflects the development of out-of-hours cooperatives in response to concerns over general practitioner (GP) workload [25]. The term “workload” was common in the keyword analysis, corroborating this hypothesis. Improvements in communication technology may have made telephone triage more accessible and easier to adopt. Around this time, integrated nursing “hotlines,” based in call centres, were set up for wide-scale provision, which gradually replaced traditional out-of-hours services. Examples of these included NHS Direct in the UK [5, 23] and Swedish Healthcare Direct [26].

Between 2019 and 2020, research output doubled. Keyword analysis using overlay maps suggests that this may have been due to the COVID-19 pandemic, with terms such as “COVID-SARS-2,” “availability of healthcare,” and “coronavirus infection” appearing more often in research. Due to the increased pressure on healthcare systems and the imposition of social distancing measures during the COVID-19 pandemic, telephone triage was used to curb the spread of the virus by minimising contact between doctors and patients. At the time of performing the analysis in December 2021, 71 papers had been published in the year of 2021, suggesting the upward trend in telephone triage research persists. The use of telephone triage is likely to continue following the COVID-19 pandemic, in recognition of its value in increasing practitioner efficiency (due to fewer physical transitions between patients) and enhancing convenience for patients [27, 28].

The second aim of the bibliometric analysis was to understand the terminology used in telephone triage research. Most research articles referred to “telephone triage.” The second most common and newest term was “remote triage,” whilst “teletriage” was the least common term, even though it appeared in the research literature earlier than “remote triage.” Although the body of research articles using alternative terminology is small, given the limited corpus of research from the last forty years, excluding these terms from the searches, the number of papers in this bibliometric analysis would decrease by 12%. To enable researchers and practitioners to locate relevant research, the authors of telephone triage research articles should move towards consistent terminology using the most prevalent term “telephone triage” in future papers. This also has implications for future bibliometric analyses and reviews in other fields, such that, inconsistent terminology should be considered and input to search engines to uncover all relevant papers, especially where research is limited, to maximise learning potential from reviews.

The third aim of the analysis was to understand where telephone triage research has been undertaken and by whom. This was achieved through the analysis of both country affiliations and author co-occurrences. Much of the research has been carried out in developed countries like the USA, UK, and Australia. This likely reflects the presence of long-standing telephone triage services (e.g., NHS Direct, NHS 111, and Healthdirect Australia) in these nations. NHS Direct and Healthdirect Australia have offered universal access to telephone triage services since the early 2000s, aiming to allocate medical resources efficiently and enhance patient convenience. These differ to the provision in the US, where insurance companies have been offering telephone triage services to incentivise patients to sign up since the 1990s [29].

Whilst evidence suggests that there are some collaborations between authors in different countries, cluster mapping revealed the most-cited authors mainly researched within the same systems. Before implementing evidence-based change in telephone triage settings, practitioners should consider the relevance of evidence to their local context. Additionally, there is a need for more research focusing on telephone triage systems in developing countries and cross-country evaluations to understand how generalisable telephone triage research is across different contexts.

The penultimate aim of this analysis was to identify specific journals in which telephone triage research was published more frequently. The journal with the most papers pertaining to telephone triage was the Journal of Telemedicine and Telecare, although the total number of publications was small (*n* = 18). Instead, telephone triage articles were dispersed across a wide range of journals, some of which were specific to particular healthcare contexts (e.g., Oncology and Pediatrics). The diverse range of journals where telephone triage research is published can make it arduous for researchers to locate relevant journal articles. However, this bibliometric analysis offers a strategy for searching, i.e., exploring specialist journals relevant to the specific healthcare context of interest.

Despite the relatively low number of research papers on telephone triage in the 1980s and 1990s, most of the highly cited papers are from this era. This may be due to time, as the older the papers are, the more likely they are to be cited [30]. Given the evolving complexity of telephone triage services in recent years, including the incorporation of technology like computer decision support software and the shifting of triage responsibilities to nonclinical staff, contemporary researchers should consider potential temporal validity issues of older research and prioritise more recent research evidence when implementing changes.

The final aim of this analysis was to obtain an overview of the research themes addressed in the telephone triage literature. This is where VOSviewer's ability to analyse keywords was particularly useful and versatile. Maps showing trends by year, as well as co-occurrences of keywords, facilitated an understanding of general themes, whilst the ability to provide text outputs enabled a brief quantitative content analysis to identify information pertaining to settings, professions, and outcomes. This was especially useful given the “noise” caused by database-indexed keywords. This helps to understand research gaps too. For example, there was significantly more research pertaining to emergency and ambulatory care, as opposed to urgent and nonurgent primary care, in which telephone triage has been widely adopted in recent years. Nonclinical staff performing triage (such as GP receptionists, or health advisors in the English NHS111 system) were rarely the subject of telephone triage research. More research should be conducted to understand the experiences of nonclinical staff and the factors influencing patient outcomes such as satisfaction and safety, in these systems [31]. Moreover, relevant to diversity and inclusion, the discovered demographic keywords were related to age and gender, as opposed to ethnicity, language, or socioeconomic status (see Figure 7 which shows keywords like “middle aged,” “male,” “female,” and “young adult”). These gaps merit further study to check the suitability of telephone triage for the diverse populations they serve, especially since language barriers have been shown to negatively affect patient safety [32]. Keywords pertaining to outcomes mainly focused on “quality”-related words, though without more in-depth analysis, it is not clear how quality was measured. Safety was the second most commonly occurring outcome-related keyword, in contrast to previous reviews suggesting that the safety of telephone triage is under-researched [9, 10]. However, these reviews focused on out-of-hours and telephone advice nursing services, so it seems reasonable to suggest that the majority of safety research has taken place in emergency care settings. A systematic review of literature relating to telephone triage in primary care is ongoing [16] and should address the mixed findings concerning the actual volume of telephone triage safety research.

### 4.1. Limitations

Bibliometric analysis has several limitations. Firstly, the quality of the output is reliant on the quality of the indexing of databases. Where many terms are indexed, this can create a substantial amount of “noise” in a cluster map; however, in the current analysis, best efforts were made to use author-indexed keywords to increase reliability. Additionally, incorrect indexing can affect the conclusions drawn from the analysis. For example, in the current analysis, using author-indexed keywords only, it may have been concluded that most research has been conducted in primary care; however, using author-indexed and VOSviewer generated (“all keywords”) revealed that the term “emergency medical services” occurred more often than primary care, enabling more specificity for interpretation. The current review supports the use of the VOSviewer natural language processing algorithms for keyword analysis, since different findings were produced by the same dataset when author and index keywords were combined (i.e., using the “all keywords” function). Moreover, to counter the subjectivity of keyword analysis, MeSH key terms (indexed using standardised headings) from a secondary database (PubMed) were used and corroborated thematic findings using standardised, indexed keywords, increasing the reliability of the authors' interpretation of the results. Researchers performing future bibliometric analyses are encouraged to do the same.

Secondly, the analysis is based on citation information taken primarily from one database, though this was justified since other databases fail to give the level of bibliometric data Scopus does, such as geographical information. Finally, a degree of subjectivity on the part of the researcher is required to make sense of cluster mapping analyses, given the lack of in-depth information relating to the citations, and the current analysis is no exception. However, it is hoped that both of these limitations will be addressed at least partially by an ongoing systematic review into the safety of telephone triage [16]. This approach is modelled on Cheng et al.'s [14] trimethod which included a bibliometric analysis, content analysis, and systematic review to understand the scope and scale of adventure tourism research. Despite these limitations, the review still provides a useful “at-a-glance” analysis of telephone triage research literature for busy researchers, students, and practitioners alike.

### 4.2. Future Research Directions and Implications

The current review has provided an overview of the telephone triage literature and proposed challenges for practitioners looking to adopt or improve their systems using evidence-based practice. Additionally, the review identifies several areas which merit further research. Firstly, reviewing the citation information revealed that much of the research was carried out in developed countries and found limited evidence of cross-country collaboration. Investigating and comparing systems in different countries may help to ascertain generalisability of research findings and support health systems to identify what might work for their healthcare context. Moreover, more detailed analysis of research taking place within countries or individual healthcare organisations would provide a greater understanding of research trends, help evaluate services, and implement improvements. Secondly, keyword analysis was particularly important for understanding the research corpus and identifying gaps in the literature. For example, keywords pertaining to care settings suggest that emergency services were most studied. Further research in other domains of healthcare and dentistry would be useful, particularly since the expansion of telephone triage and home care services during the COVID-19 pandemic [33]. Review of keywords pertaining to professional role mainly focused on doctors and nurses triaging patients. Telephone triage activities have shifted to nonclinical staff, such as NHS 111's health advisors, and GP receptionists; therefore, attention should be given to the evaluation of these services, to understand their impact on patient outcomes and implement evidence-based practice to improve. Finally, keywords relating to outcomes focused mainly on quality and safety of telephone triage. More detailed reviews of these constructs would be useful to understand research methods used and quantify these outcomes. An upcoming systematic review by this author group is aimed at addressing this by reviewing the safety literature available from the same databases.

### 4.3. Applications for Healthcare Systems

Further to the research requirements outlined above, there are several applications for healthcare systems arising from this analysis. Firstly, this analysis serves as an accessible evidence summary for those working in healthcare implementation and quality improvement, where time and resources restrict the ability of healthcare personnel to conduct evidence synthesis. Secondly, the analysis has outlined the importance of considering the amount and relevance of evidence relating to specific healthcare contexts prior to implementation or system change. This should enable those working in healthcare quality improvement or project management to consider the most relevant evidence for their setting and help prioritise areas for evaluation. For example, the paucity of literature pertaining to nonclinical triage professionals makes the case for careful piloting of these types of workers, prior to implementation. The research gaps addressed in this analysis could also be used to support decisions about the allocation of research funding and other resources in organisations using or implementing telephone triage. Finally, healthcare practitioners using telephone triage should reflect on the paucity of evidence relating to demographic information such as ethnicity, language, or socioeconomic status and carefully consider how these might affect telephone triage quality and safety in their practice and wider organisation.

## 5. Conclusions

This bibliometric analysis provides a macroscopic overview for researchers, practitioners, and policymakers interested in telephone triage research or implementation. This is important since telephone triage has become increasingly commonplace in modern healthcare. The analysis highlights a paucity of peer-reviewed literature over the last forty years, particularly in nonemergency settings (though this is growing since the outbreak of the COVID-19 pandemic), creating a case for further research. Additionally, the review identified obstacles which make it difficult for researchers to identify relevant research including lack of consistency in terminology used by authors. By providing contextual information about research settings, the review challenges and provides guidance to those with an interest in evidence-based practice, to consider the generalisability of the research evidence to their individual settings before implementing change. Moreover, it provides a useful example of bibliometric analysis, along with a discussion of its merits and limitations, which should be helpful to those with an interest in the method (e.g., systematic reviewers or early career researchers seeking to address gaps in a field to direct further research), especially since no reporting protocols (like those available for systematic reviews) currently exist.

## Figures and Tables

**Table 1:** Number of articles retrieved from searches after exclusion of future papers.

Database	Total number of articles identified for all search terms	Median publications per year
Scopus	784	20.5
PubMed	679	N/A

**Table 2:** Trends in the usage of telephone triage terminology, including publication year and percentage of research output.

Terminology used	Number of identified articles	First publication year	Percentage of total research output
Telephone triage	691	1980	88.1%
Teletriage	43	1981	5.5%
Remote triage	50	1997	6.4%
Total	784	—	—

**Table 3:** Journals with ten or more telephone triage research articles and their corresponding impact factor according to Scopus CiteScore (accurate as of October 2022).

Source title	Number of publications	Scopus CiteScore
Journal of Telemedicine and Telecare	18	11.9
British Journal of General Practice	18	4.2
BMJ Open	17	3.9
Pediatrics	11	N/A
Huisarts en Wetenschap	11	0.1
Journal of Advanced Nursing	10	4.3
Family Practice	10	3.3
BMC Family Practice	10	N/A

N/A means that information was unavailable.

**Table 4:** Ten most cited papers in telephone triage research according to Scopus database (all terminologies included).

Authors	Year	Title	Cited by	Journal
Bunn et al.	2004	Telephone Consultation and Triage: Effects on Health Care Use and Patient Satisfaction	180	Cochrane Database of Systematic Reviews (online)
Diehr et al.	1984	Prediction of Pneumonia in Outpatients with Acute Cough—A Statistical Approach	158	Journal of Chronic Diseases
Haley	2003	Family Caregivers of Elderly Patients with Cancer: Understanding and Minimizing the Burden of Care	136	The Journal of Supportive Oncology
Leibowitz et al.	2003	A Systematic Review of the Effect of Different Models of After-Hours Primary Medical Care Services on Clinical Outcome, Medical Workload, and Patient and GP Satisfaction	131	Family Practice
Poole et al.	1993	After-Hours Telephone Coverage: The Application of an Area-Wide Telephone Triage and Advice System for Pediatric Practices	123	Pediatrics
Gulliford et al.	1997	Popularity of Less Frequent Follow Up for Breast Cancer in Randomised Study: Initial Findings from the Hotline Study	122	British Medical Journal
Christensen and Olesen	1998	Out of Hours service in Denmark: Evaluation Five Years after Reform	113	British Medical Journal
Campbell et al.	2014	Telephone Triage for Management of Same-Day Consultation Requests in General Practice (the ESTEEM Trial): A Cluster-Randomised Controlled Trial and Cost-Consequence Analysis	112	The Lancet
Leprohon and Patel	1995	Decision-Making Strategies for Telephone Triage in Emergency Medical Services	110	Medical Decision Making
Giesen et al.	2007	Safety of Telephone Triage in General Practitioner Cooperatives: Do Triage Nurses Correctly Estimate Urgency?	94	Quality and Safety in Health Care

**Table 5:** A table showing where telephone triage research has been undertaken, by geographical continent, including percentage dominance of published literature.

Continent	Number of publications	Percentage dominance
Europe	353	45.0%
North America	298	38.0%
Oceania	70	8.9%
Asia	47	6.0%
South America	5	0.6%

**Table 6:** Countries with the highest number of publications in telephone triage, according to the Scopus database.

Country	Number of documents in Scopus
United States	263
United Kingdom	160
Australia	63
Netherlands	49
Canada	35
Denmark	29
Italy	22

**Table 7:** Content analysis of keywords relating to telephone triage setting, professional doing triage, and outcome of study, with more than 20 occurrences.

Setting	Number of occurrences	Profession	Number of occurrences	Outcome	Number of occurrences
Ambulance/ambulatory care/emergency care/emergency health service/emergency medical services	625	General practitioner(s)	118	Health care quality/quality of healthcare	104
Primary care/primary health care/primary medical care	191	Nurse(s)/nursing staff	133	Patient safety/safety	59
After-hours care/out-of-hours care	124	Physician	28	Patient satisfaction	58
Emergency service hospital/hospital emergency service	92	Nurse practitioner	22	Utilization (review)	53
Hotlines	88			Health services accessibility	38
General practice	73			Outcome assessment	37
Emergency ward	53			Decision-making	37
Pediatrics	37			Workload	36
Family practice	36			Nursing assessment	36
Home care	21			Time factors	32
Pediatric nursing	20			Interpersonal communication	27
				Sensitivity and specificity	27
				Clinical competence	25
				Attitude of health personnel	22
				Patient attitude	22
				Nurse attitude	21
				Patient compliance	21
				Risk assessment	21
				Nurse-patient relations	20

## Data Availability

The citation information supporting this bibliometric analysis is from previously reported studies and is available using the search criteria and databases outlined in the manuscript. The processed data are available from the authors upon request.

## Abbreviations

COVID-19: Coronavirus disease of 2019
CSV: Comma separated values
GP: General practitioner
ICD-10: International Statistical Classification of Diseases and Related Health Problems
MeSH: Medical Subject Headings
NHS: National Health Service
OOH: Out-of-hours
SARS-COV-2: Severe acute respiratory syndrome coronavirus 2
SNOMED-CT: Systematized Nomenclature of Medicine Clinical Terms
VOS: Visualisation of similarities.
